# A Vision-Guided Robotic System for Safe Dental Implant Surgery

**DOI:** 10.3390/jcm13216326

**Published:** 2024-10-23

**Authors:** Daria Pisla, Vasile Bulbucan, Mihaela Hedesiu, Calin Vaida, Ionut Zima, Rares Mocan, Paul Tucan, Cristian Dinu, Doina Pisla, TEAM Project Group

**Affiliations:** 1 Department of Maxillofacial Surgery and Radiology, Oral Radiology, “Iuliu Hatieganu” University of Medicine and Pharmacy, 32 Clinicilor Street, 400006 Cluj-Napoca, Romania; daria.pisla@elearn.umfcluj.ro (D.P.);; 2 CESTER—Research Center for Industrial Robots Simulation and Testing, Technical University of Cluj-Napoca, 28 Memorandumului Street, 4000114 Cluj-Napoca, Romania; vasile.bulbucan@mep.utcluj.ro (V.B.); calin.vaida@mep.utcluj.ro (C.V.); doina.pisla@mep.utcluj.ro (D.P.); 3 Department of Maxillofacial Surgery and Radiology, Maxillofacial Surgery and Implantology, “Iuliu Hatieganu” University of Medicine and Pharmacy, 37 Iuliu Hossu Street, 400429 Cluj-Napoca, Romania

**Keywords:** dental implants, robotic-assisted oral surgery, guided surgery, navigation surgery

## Abstract

**Background:** Recent advancements in dental implantology have significantly improved outcomes, with success rates of 90–95% over a 10-year period. Key improvements include enhanced preplanning processes, such as precise implant positioning, model selection, and optimal insertion depth. However, challenges remain, particularly in achieving correct spatial positioning and alignment of implants for optimal occlusion. These challenges are pronounced in patients with reduced bone substance or complex anthropometric features, where even minor misalignments can result in complications or defects. **Methods:** This paper introduces a vision-guided robotic system designed to improve spatial positioning accuracy during dental implant surgery. The system incorporates advanced force-feedback control to regulate the pressure applied to bone, minimizing the risk of bone damage. A preoperative CBCT scan, combined with real-time images from a robot-mounted camera, guides implant positioning. A personalized marker holder guide, developed from the initial CBCT scan, is used for patient–robot calibration. The robot-mounted camera provides continuous visual feedback of the oral cavity during surgery, enabling precise registration of the patient with the robotic system. **Results**: Initial experiments were conducted on a 3D-printed mandible using a personalized marker holder. Following successful patient–robot registration, the robotic system autonomously performed implant drilling. To evaluate the accuracy of the robotic-assisted procedure, further tests were conducted on 40 identical molds, followed by measurements of implant positioning. The results demonstrated improved positioning accuracy compared to the manual procedure. **Conclusions**: The vision-guided robotic system significantly enhances the spatial accuracy of dental implants compared to traditional manual methods. By integrating advanced force-feedback control and real-time visual guidance, the system addresses key challenges in implant positioning, particularly for patients with complex anatomical structures. These findings suggest that robotic-assisted implant surgery could offer a safer and more precise alternative to manual procedures, reducing the risk of implant misalignment and associated complications.

## 1. Introduction

A dental implant is a medical device surgically placed into the jawbone or skull to be used as a replacement for the root of a missing tooth. The implant serves as a support for a dental prosthesis (crown, facial prosthesis) or as an orthodontic anchor [1]. The most recent statistical data from 2024 show that the success rate of dental implants varies between 90% and 98% over a period of 10 years [2,3]. There are several factors that influence the success rate of dental implants [4]: implant location, patient health, bone quality, surgical expertise, and oral hygiene [5].

While the overall success rate of dental implants is quite high, there are still specific challenges that require improvements. The most important one refers to the manual positioning accuracy of the implant, which, in specific cases, must be improved. Recent studies show that advanced techniques like static and dynamic computer-assisted implants [6], positional guides [7], and dynamic navigation [8] show results of 0.85–1.29 mm (static navigation techniques) and 0.37–1.34 mm (dynamic navigation techniques) in horizontal deviation at the entry point; 1.18–1.83 mm (static navigation technique) and 0.38–1.52 mm (dynamic navigation techniques) in horizontal deviation at the implant apex; and 1.6–6.46 degrees (static navigation techniques) and 0.89–4.45 degrees (dynamic navigation techniques) in angular deviation.

As demonstrated in the literature, manual dental implants offer the advantages of shorter procedural times and cost-effectiveness but come with risks of human error and accuracy limitations. While this technique allows experienced dentists to effectively use their skills, the variability in training and experience can impact the success rates and predictability of the results [6,9].

A revolutionary approach in dental implantology may be considered robotic assistance in surgical procedures. Robotic systems may enhance the placement accuracy of the implant, minimize the complications, and maximize the stability and lifetime of the implant [10]. By using preoperative planning and real-time feedback during the procedure, robotic systems can reduce the risk of damaging surrounding anatomical structures such as nerves and blood vessels [11,12,13]. Robotic systems may also use advanced imaging techniques to create detailed surgical plans, providing predictability and ensuring consistent outcomes for different patients, thus reducing the variability associated with the manual procedure [14]. By guiding the surgeon’s movement, a robotic system can help mitigate risks associated with human error, leading to more reliable and accurate implant placement [15]. The use of a robotic system can also assist less experienced surgeons in performing complex procedures with increased accuracy. All the above advantages make robotic systems an increasingly popular solution in implantology.

A recent literature review published by Bahrami et al. [16] divides robotic solutions for dental implantology into three categories: active robots that can enter and exit the oral cavity of the patient autonomously, prepare the site and place the implant; passive robots that require the operator to guide the robotic arm during the procedure with no (zero) autonomy; and semi-active robots that can autonomously perform the implant but require operator guidance during entry/exiting the oral cavity.

An in vitro study [17] comparing the precision of various human–robot interaction modes revealed that active robotic systems outperform passive robots, offering superior accuracy and significantly reducing operation time. These findings emphasize the potential of robotics to enhance dental implant procedures. However, despite the enhanced precision and efficiency offered by active systems, human collaboration remains essential, as complete automation in complex surgeries like dental implant placement is still not entirely possible. While robots reduce the margin for error, they are not immune to malfunctions or misalignments, requiring constant monitoring and adjustments by trained professionals. In another study investigating autonomous robotic systems for dental implant placement [18], the results demonstrated higher accuracy than traditional manual procedures, with a mean global coronal deviation of 0.74 mm and an angular deviation of 1.11°. These figures suggest that autonomous systems can significantly improve implant placement precision, potentially reducing complications and improving patient outcomes. However, this increased accuracy comes with its own set of challenges, such as the potential for system errors, which could compromise results if not adequately managed. Furthermore, the reliance on robotic systems introduces a learning curve for practitioners, who must become proficient in operating these advanced machines to fully benefit from their capabilities. The Yomi system [19], developed by Neocis, is the first FDA-approved robotic assistant specifically designed for dental implant surgery. It combines robotic technology with the expertise of dental surgeons to ensure more precise implant placements. Yomi allows for detailed preoperative planning using 3D imaging while also providing haptic feedback during the procedure, enabling surgeons to maintain control and make real-time adjustments. This blend of automation and human skill helps reduce the risks associated with manual procedures, such as deviations in implant positioning or excessive tissue damage. However, despite these advantages, the introduction of robotic systems like Yomi requires significant investment in both technology and training.

Summarizing the data published in the scientific literature cited above, there is a strong motivation for new research that aims to improve the outcome of dental implants.

As a possible response to the challenges presented in the introduction, this paper proposes the use of an innovative robotic system to actively perform dental implant surgical procedures [20].

The specific objectives proposed within this research are the achievement of a higher spatial positional accuracy of the implant, especially with respect to angular deviation to ensure efficient occlusion, the alignment improvement of multiple implants, and the successful positioning of implants for patients with poor bone structure or challenging anatomy.

## 2. Materials and Methods

The robotic system proposed within this research consists of a collaborative robotic arm, a 3D navigation system based on a 3D camera, and a control system integrating means for controlling the robot, the camera, and the physiodispenser under surgeon supervision. The robot arm is equipped with torque sensors, which enable efficient real-time control of the forces and torques during the drilling procedure, allowing autonomous drilling and tapping for the dental implant. The robotic system uses real-time visual feedback from an end-effector mounted camera, enabling the mapping of the oral cavity over the CBCT scans to dynamically adjust the end-effector position by monitoring patient head movements. The real-time torque monitoring capabilities are used to maintain an efficient feed of the drill/tapper during drilling/tapping adapted to the bone density. The robot is controlled using the ROS (Robot Operating System-Open Robotics, San Francisco, CA, USA) environment, which integrates motion control, camera feedback, and torque control/monitoring while providing, at the same time, a visual interface that displays the digital twin of the robot. The preplanning procedure is performed using a baseline CBCT (Cone Beam Computer Tomography) of the patient, where the position of the implant is established by the surgeon and then dynamically registered with respect to a visual marker used by the robot camera for real-time adjustments during the procedure. For the robot–patient registration, a 3D-printed marker holder guide is used to accurately align the implant spatial position with the visual marker. The guide is designed to be attached in a position that does not hinder the implant surgery, and using the latest 3D printing technologies is achieved as a single printed assembly with parts of different materials (a hard material for the marker support and a soft, elastic one, for the guide attached to the mandible). Using the pre- and intraoperative data, the robotic system is able to perform autonomously the drilling and tapping of the implant.

### 2.1. The Medical Protocol for Dental Implant Procedure

**Figure 1:**
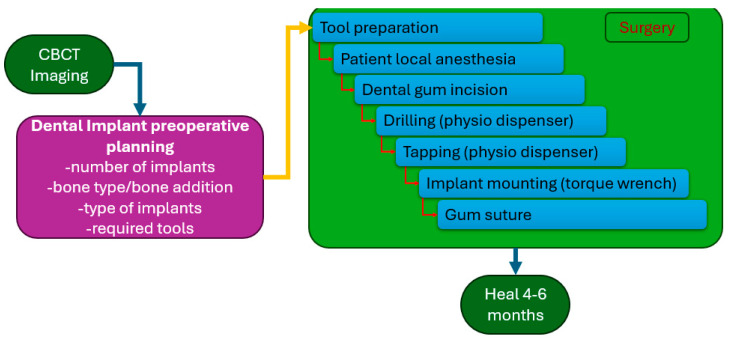
Medical protocol for the manual dental implant procedure for bone-level implant.

Based on surgeons’ information regarding the manual dental implant procedure, a medical protocol was developed and presented in Figure 1.

For this initial stage of the research, the bone-level implant was considered, which is the most common type of dental implant. In this procedure, the implant is placed at the level of the alveolar bone, allowing flexibility and aesthetic advantages. Bone-level implants are positioned in such a manner that the implant platform is at the level of the alveolar bone or slightly under, allowing soft tissue to heal over the implant. A two-piece design of the implant was considered, consisting of the implant body (embedded in the bone) and the abutment attached to the implant body after the healing phase. The insertion technique considered was a two-stage technique, meaning that the implant is placed at the bone level, covered by soft tissue, and allowed to heal before performing a second surgery in order to expose the implant’s platform and mount the abutment.

The surgical procedure is preceded by the preplanning phase, where, with the help of a CBCT scan, several procedural parameters are determined:

- The number of implants (based on the gap provided by missing teeth or an orthodontic device that is going to be mounted on the implant);
- The bone structure analysis and, if necessary, bone addition;
- The spatial position of the implant(s);
- The implant(s) type and tools required for the procedure.

Drilling for the implant is performed using a physiodispenser that is able to spin the tools at lower speeds than usual milling devices to avoid bone fracture. The surgical procedure begins with patient anesthesia performed locally. After the anesthesia, the gum is incised, and the first hole is drilled using a spear mill (this step is called cortical perforation). The hole diameter is increased using successive drills with larger sizes until the nominal diameter for the implant thread is achieved. The implant hole is threaded, followed by the implant screwing using a dental torque wrench. The implant is covered with a protective screw, and the gum is sutured. The healing time for this dental implant protocol is between 4 and 6 months, which is necessary to allow the osseointegration of the implant.

**Figure 2:**
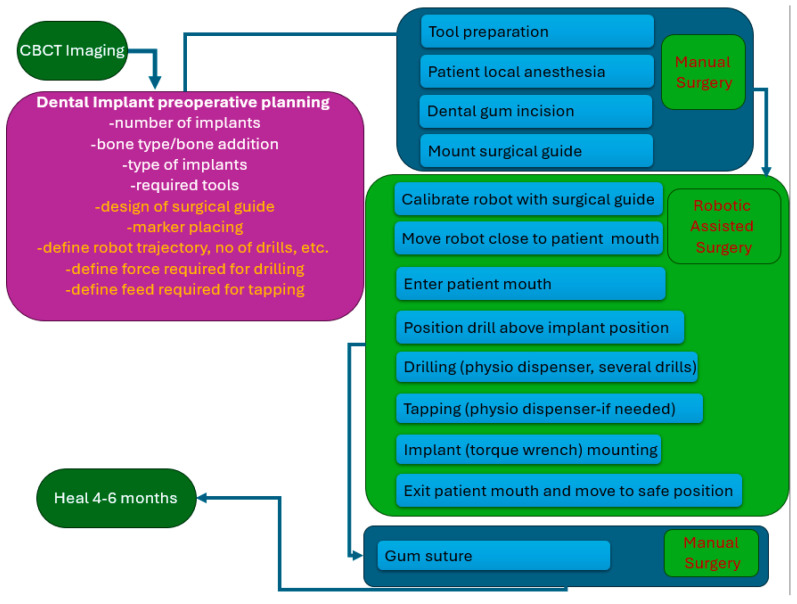
Protocol for robotic-assisted dental implant procedure.

**Figure 3:**
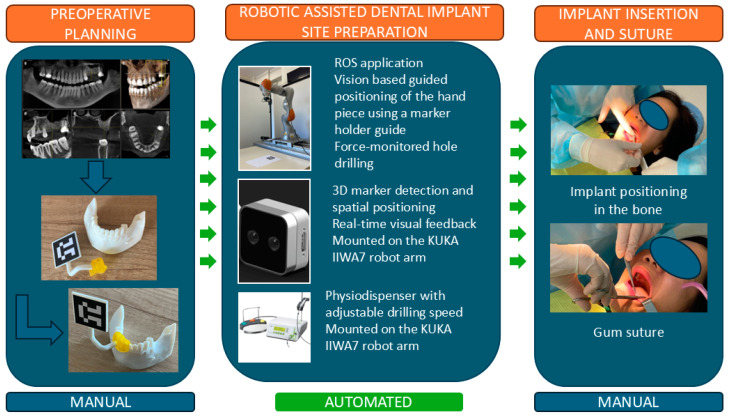
Manual and automated stages in robotic-assisted implant surgery.

Based on the protocol from the manual procedure, a new protocol for the robotic-assisted procedure was developed, as illustrated in Figure 2. The preplanning phase contains several additional steps required to allow the robot to autonomously perform the drilling and tapping of the implant site. A marker holder guide is designed and 3D printed as a single part using biocompatible materials to allow the accurate positioning of the marker for the 3D camera used in robot navigation, which is required for the robot–patient registration (a detailed description of the guide is presented in Section 2.2). The coordinates of the implant screw required for the drilling are computed, and the robot trajectory is defined together with the number of drills required for the nominal diameter of the implant hole. Based on the determined bone density, the dynamic parameters for the procedure are defined: the optimum force and rotation speed during the drill, followed by the feed for tapping the implant hole. The medical protocol for robotic-assisted dental implant surgery still contains several procedural steps that require the intervention of the surgeon: preparation of the tools required for the procedure, the anesthesia of the patient, gum incision, and the positioning of the guide with the visual marker. The surgeon performs the robot calibration using the visual marker, and upon validation, the next steps are performed autonomously by the robot under the surgeon’s supervision. The robot performs the number of drills defined in the preplanning phase and the threading of the implant hole, and the surgical procedure is finished by the surgeon by mounting the implant and suturing the gum. A detailed description of the manual and automated steps is shown in Figure 3.

### 2.2. Preoperative Planning

**Figure 4:**
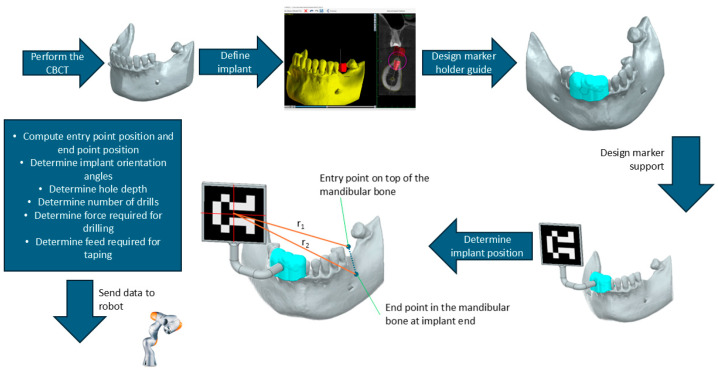
Preplanning of the robotic-assisted dental implant procedure.

The preplanning phase contains several important procedural actions. Figure 4 presents the steps required before the surgery to obtain the input data for the robot.

➢ Perform the CBCT: The preoperative planning starts by performing a CBCT scan, which is used to generate a 3D image of the jawbone, allowing the surgeon to achieve a detailed view of the patient’s oral cavity.
➢ Define the implant: Once the CBCT data are available, the next step is to define the implant position and orientation. This step involves selecting where the implant will be placed and determining its proper angle, depth, and alignment within the bone. Figure 4 shows the implant in red color, indicating its desired position on the jawbone.
➢ Design of the marker holder guide: During this step, a guide for the video camera marker is designed. This guide uses the 3D model of the jawbone to perfectly be attached to 3 or more teeth. This guide is generated similarly to a classical surgical guide, but it does not contain a guiding hole for the drill; its purpose is to create a reference system between the 3D camera marker and the site of the implant.
➢ Design of marker support: The navigation system requires several markers in order to dynamically adjust the position of the robot with respect to the implant site. This system uses a single marker that allows the determination of the position and orientation of the robot’s end-effector with respect to the marker. The marker is placed on a special plate attached to the holder guide. By determining the position of the robot with respect to the camera marker and, at the same time, knowing the position of the implant site with respect to the same marker, the robot can be registered to the implant site.
➢ Determine implant position: The position of the implant site with respect to the camera marker is determined using specialized software. In this stage, the orientation angles and the depth of the implant are extracted, and the surgeon provides a list of necessary tools (drills, tapers, wrenches) to be used by the robot during the procedure, based on bone analysis, the density of the bone is determined, and the force and speed required for each drill are computed.

Ways to determine the data required for the robotic procedure in the preoperative planning phase are provided in the following lines.

**Figure 5:**
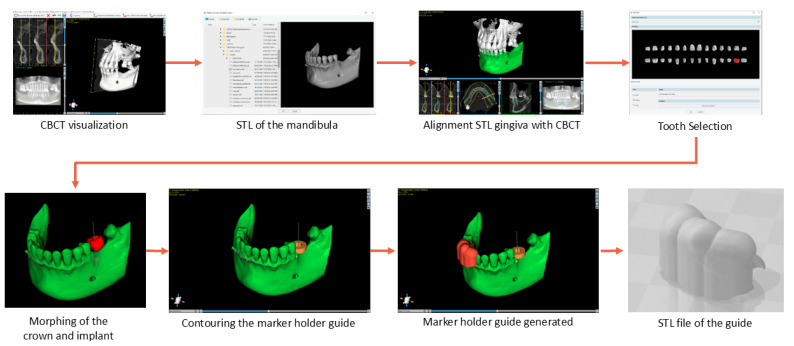
Design and manufacturing of the marker holder guide.

**Figure 6:**
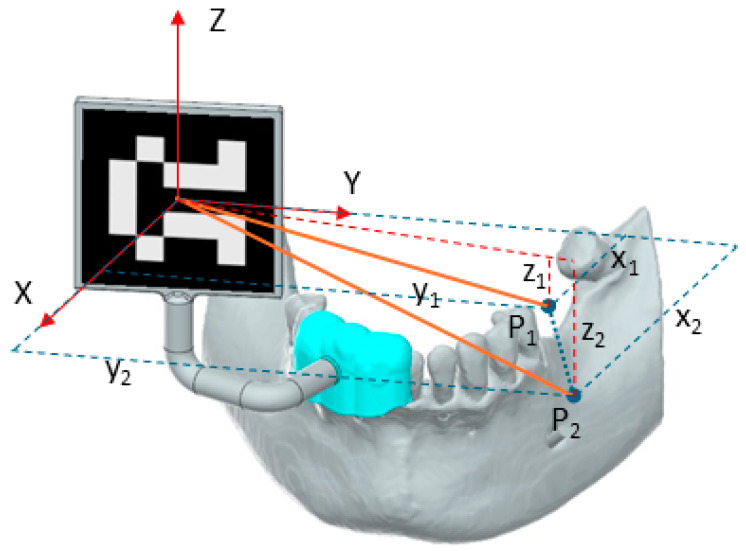
Starting point and ending point for the drilling with respect to marker position.

The marker’s guide is obtained using Blue Sky Plan 4 [21] software following the steps in Figure 5. A segmentation of the lower base of the mouth is required for the software to align the STL resulting from the segmentation with the position of the teeth in the CBCT. Once the STL is imported into the program, the alignment is performed automatically, but it still needs manual fine positioning for accurate placement. After the proper alignment, the tooth that will use the implant is selected, and the software places the implant in the selected position. The maker holder guide is obtained by selecting several contour points and is generated based on these points. Using the generated guide, an extension required for marker mounting is also designed and attached to the guide, which results in an assembly of a soft, elastic part (the guide) and a hard part, the marker support. The marker support is designed as a fixed attachment to the guide and through a bent thin pipe to extend outside the oral cavity where the visual marker is positioned, enabling the robot to permanently adjust its position relative to its spatial location. Following the design of the marker extension and the placing of the video marker, the coordinates of the implant points of interest (*P*_1_ and *P*_2_) are defined. The determination of the coordinates is illustrated in Figure 6. These values can be directly measured within the implant planning software or can be extracted from the 3D modeling software (Siemens NX 2206 Series). The 3D Cartesian system is defined as aligning the ZOY plane to the coronal plane, the ZOX plane to the sagittal plane, and the XOY plane to the axial plane. To compute the motion trajectory of the robot, the values of X_1_, X_2_, Y_1_, Y_1_, Z_1_, and Z_2_ coordinates have to be known.

The data required for the robotic system to compute the trajectory of the robot and the drilling parameters are provided in Equation (1):

(1) $$\left\{\begin{array}{l} P_{1}(x_{1},y_{1},z_{1})\\ P_{2}(x_{2},y_{2},z_{2})\\ \rho \end{array}\right.$$

Points *P*_1_ and *P*_2_ are described above and represent the insertion and target point of the robot tool (the points of interest determined from the CBCT scan). The ρ parameter defines the bone density in the drilling area, data that are extracted from the CBCT files.

To compute the implant orientation with respect to the video marker position, the coordinates of points *P*_1_ and *P*_2_ are used, along with the direction cosines, enabling the computation of the orientation of the line that connects point *P*_1_ with *P*_2_ with respect to the reference system axes.

First, the length of the $P_{1}P_{2}$ segment is determined using Equation (2):

(2) $$P_{1}P_{2}=\sqrt{{\left(x_{2}-x_{1}\right)}^{2}+{\left(y_{2}-y_{1}\right)}^{2}+{\left(z_{2}-z_{1}\right)}^{2}}$$

Using Equation (2), the cosine angles *θ_x_*, *θ_y_*_,_ and *θ_z_* between the vector and the *x*, *y*, and *z* axes are computed:

(3) $$\left\{\begin{array}{l} \cos\theta_{x}=\frac{x_{2}-x_{1}}{\left|\vec{P_{1}P_{2}}\right|}\\ \cos\theta_{y}=\frac{y_{2}-y_{1}}{\left|\vec{P_{1}P_{2}}\right|}\\ \cos\theta_{z}=\frac{z_{2}-z_{1}}{\left|\vec{P_{1}P_{2}}\right|} \end{array}\right.$$

This yields the angles from Equation (4):

(4) $$\left\{\begin{array}{l} \theta_{x}=\arccos\left(\frac{x_{2}-x_{1}}{\left|\vec{P_{1}P_{2}}\right|}\right)\\ \theta_{y}=\arccos\left(\frac{y_{2}-y_{1}}{\left|\vec{P_{1}P_{2}}\right|}\right)\\ \theta_{z}=\arccos\left(\frac{z_{2}-z_{1}}{\left|\vec{P_{1}P_{2}}\right|}\right) \end{array}\right.$$

Using the bone density parameter, the drilling feed is computed. There are several parameters that need to be defined before further processing:

― Bone density (*ρ*): The density of the bone material [g/cm^3^];
― Feed rate (*f*): The rate at which the drill advances into the material, [mm/min];
― Speed (*N*): The rotational speed of the drill, measured in RPM (revolutions per minute);
― Cutting force (*F*): The force exerted by the drill on the bone, [N];
― Drill diameter (*D*): The diameter of the drill bit, [mm].

Drilling parameters such as feed rate and speed must be adjusted based on the mechanical properties of the bone. Higher-density bones typically require lower feed rates and speeds to prevent excessive heat generation and damage. The feed rate based on bone density is computed using Equation (5), where

- *F* is the adjusted feed rate [mm/min];
- *f_base_* is the base feed rate for a reference bone density [mm/min];
- $\rho_{bone}$ is the measured bone density [g/cm^3^];
- $\rho_{ref}$ is the reference bone density [g/cm^3^];
- *n* is an empirical exponent used to adjust the sensitivity of the feed rate to changes in bone density. The value of *n* may vary based on the specific drilling equipment and clinical scenario. It typically ranges from 0.3 to 0.7 in practice [22,23].

(5) $$F=f_{base}\cdot {\left(\frac{\rho_{bone}}{\rho_{ref}}\right)}^{n}$$

Misch [24] provides a classification of bone density) into 4 categories:

― D1—dense cortical bone usually found in the mandibula (>1.25 g/cm^3^);
― D2—porous cortical and trabecular bone (0.95–1.25 g/cm^3^);
― D3—porous cortical (thin) and fine trabecular bone (0.55–0.95 g/cm^3^);
― D4—fine trabecular bone (<0.55 g/cm^3^).

Average values for maxillary bone density can range from approximately 0.3 to 0.8 g/cm^3^ in medical CBCT scans, and the density of the mandibular bone (lower jaw) tends to be higher than that of the maxilla. A more straightforward approach to computing the feed rate with respect to bone density is provided in Equation (6), where *F* is the feed rate, *N* is the revolving speed of the drill (revolutions/min), *D* is the diameter of the drill (mm) and $\rho_{a}$ is the adjusted bone density with respect to material properties.

(6) $$F=\frac{N\cdot D}{\rho_{a}}$$

Using Misch classification, several parameters of Equation (6) can be defined as shown in Table 1, and these values are determined from practical experiments. These data, substituted in Equation (6), will provide an approximate value for the feed rate during the drilling.

The threading feed in the dental implant surgery must also be determined to allow accurate placement of the implant. This step also requires high precision because the spatial alignment of the implant depends on the accuracy of the threading; a tilted thread through a correctly drilled hole leads to procedure failure. The threading feed can be determined using Equation (7), where *F_t_* is the threading feed [mm/min], *P* is the thread pitch [mm], and *N* is the dental handpiece speed during the threading operation [revolutions/min].

(7) $$F_{t}=P\cdot N$$

### 2.3. Robotic-Assisted Dental Implant Site Preparation

**Figure 7:**
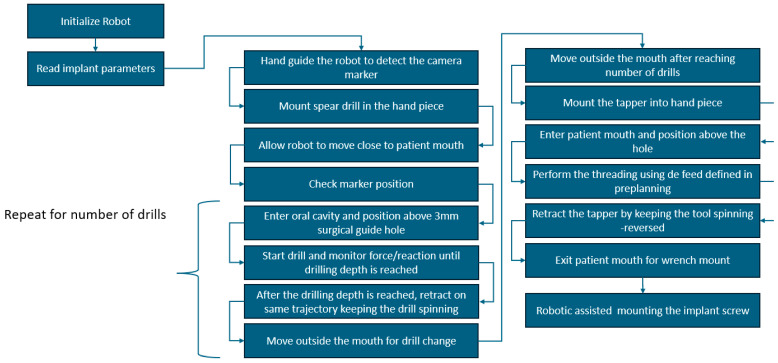
Steps of robotic-assisted oral surgery.

Following the preplanning phase, after the robotic system is initialized, the surgical procedure begins (Figure 7). At this point, the robotic system has the handpiece and the 3D camera mounted on its end-effector. The collaborative robot has hand-guiding capabilities, and the surgeon will move the robot by hand to a predefined (approximate) position from where the vision system can identify the video marker placed on the guide. After identifying the marker position, the robotic system requests the first tool (the spear drill for cortical perforation), which is mounted by the surgeon in the dental handpiece and confirmed. By acknowledging the tool attachment, the surgeon allows the robot to begin the surgical procedure. The robot will move, using real-time visual feedback, close to the oral cavity of the patient, validating once more the position of the camera marker. Before entering the oral cavity, the robotic system waits for surgeon supervision, which must be confirmed on the interface. After the robot receives surgeon validation, it enters the oral cavity and positions the surgical tool 3 mm above the insertion point (*P*_1_) using the orientation angles computed in Equation (4). This will, in fact, position the drilling tool on the line defined by the two points of interest (*P*_1_*P*_2_).

The surgeon actuates the physiodispenser set to drilling speed by pressing the foot pedal, and the drilling procedure begins. The robot moves on a linear trajectory (defined by the line connecting the two points of interest) toward the insertion point. The insertion point is reached when a force condition is raised within the robot controller, which remains raised during the entire drilling procedure. The instantaneous drilling force is continuously monitored to remain within predefined limits during the entire procedure. The drilling force, in this stage of the development, was determined empirically from several studies regarding drilling forces in different bone densities (D1 type: 30–50 N, D2, and D3 type: 20–40 N, D4 type: 10–20 N) [25,26,27]. After the drilling depth is reached, the robot retracts the dental handpiece on the same trajectory with the drill still revolving until the tool reaches the starting location above the insertion point. The robot moves outside the oral cavity, where the surgeon places the next drill in the dental handpiece, and the procedure is repeated until the threading diameter is reached. After reaching the threading diameter, the surgeon mounts the tapper into the dental handpiece, and the robot performs the threading of the implant hole, repeating the steps from the drilling phase. When the threading of the implant cavity is finished, the robot moves outside the oral cavity, the surgeon mounts the torque wrench for the implant screw, and the robot screws the implant. The procedure is finalized by the surgeon, who checks the positioning of the implant and sutures the affected area.

### 2.4. Experimental Setup

**Figure 8:**
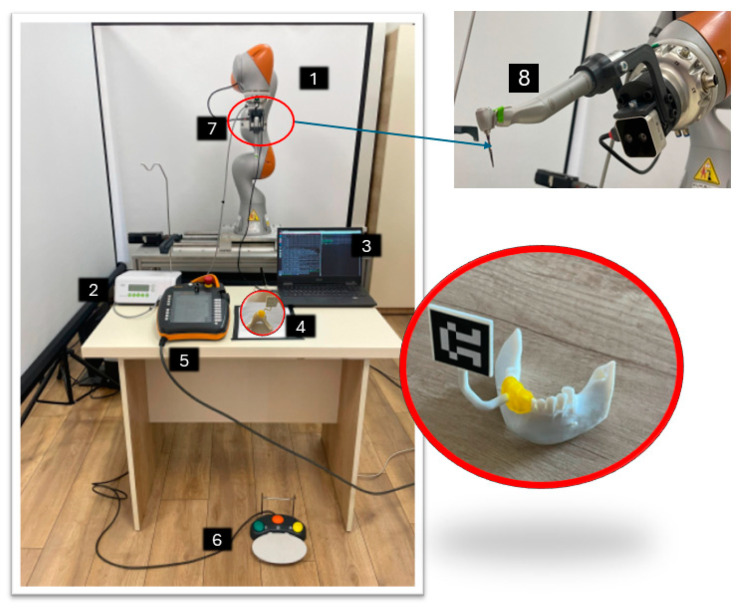
Experimental setup for robotic-assisted dental implant surgery.

The robotic system for robotic-assisted dental implant surgery proposed within this work is presented in Figure 8. The experimental setup integrates the Kuka LBR iiwa 7 R800 (1) collaborative robot from Kuka AG (Augsburg, Germany) [28] equipped with a specially designed end-effector able to incorporate the tools required for surgery and navigation. The surgical procedure is performed using a physiodispenser (2) with a dental handpiece (8) mounted in the robot end-effector. The entire process is controlled using a computer (3) that runs ROS, and the smart PAD (5) of the robot is used to start the communication server between the Kuka Sunrise Cabinet and the computer. The physiodispenser is actuated using a pedal (6), and the navigation of the robot toward the drilling area is made using an Intel RealSense D405 3D Camera (Intel Corporation, Santa Clara, CA, USA) (7) [29].

**Figure 9:**
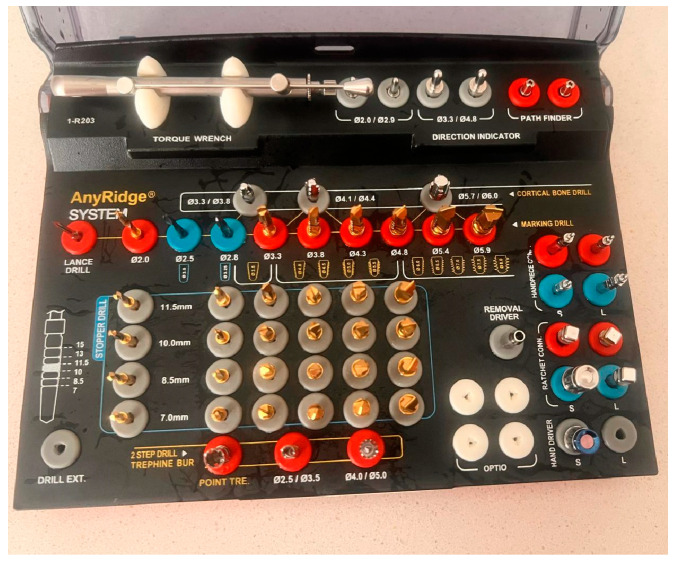
The kit for Megagen Anyridge implants.

A 3D-printed mandibula and a dedicated guide (4) are used for the experimental tests of the robotic system. The 3D-printed mandibula and the guide with the marker holder are presented in Figure 9, along with their assembled setup. The material used for the 3D samples was DraftWhite MED857 (Stratasys Ltd., Eden Prairie, MN, USA), a polypropylene commonly used in medical and healthcare applications due to its compliance with specific ASTM (American Society for Testing and Materials) standards (ASTM F748 [30], ASTM D638 [31], ASTM F1980 [32], ASTM D4101 [33]) and ISO 10993 [34], the characteristics of the sample are available at [35]. The experimental setup is configured to perform the pilot drill of the implant followed by sequential drilling using different diameters drill depending on the implant size (for the experimental test presented within this paper, a 4.5 mm diameter and 10 mm length implant was used requiring two sequential drills one with a 2.8 mm drill and the other one with a 3.3 mm drill) due to low-density material of the 3D-printed mandibula (Type D4) there was no tapping of the hole implant required, but the system is capable of performing the implant site for different types of implant, in this case, the implant tool kit for Megagen Anyridge [36] implant presented in Figure 9 was used.

**Figure 10:**
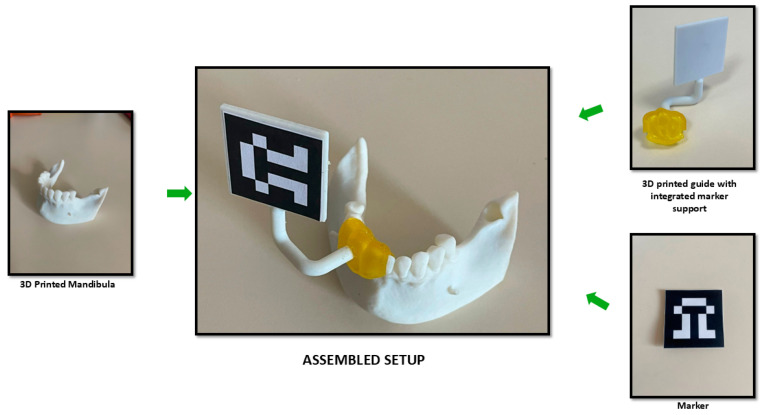
Three-dimensional printing of the dedicated guide and placing of the marker.

As can be seen in Figure 10, the assembly consisting of the guide and the marker support is printed as a single 3D component, which ensures no placement errors between the guide and the marker holder.

### 2.5. Robot Control

**Figure 11:**
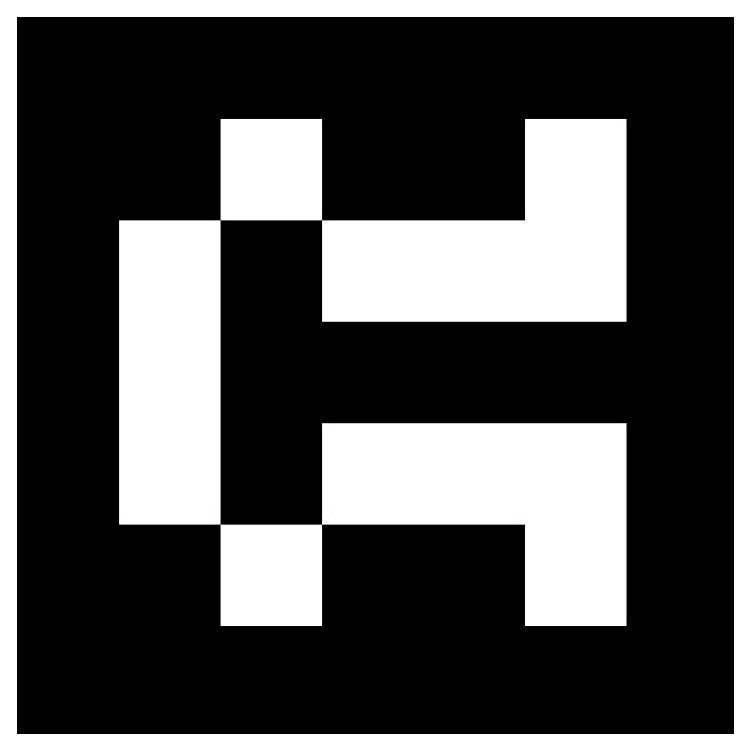
Generated marker.

The robot is controlled during the surgical procedure using the ROS environment [37,38]. The ROS environment integrates the stereo (3D) camera, facilitating autonomous navigation and orientation for the dental procedure experiment. By integrating the stereo camera, the primary objective is to enable the robot to identify designated markers within the environment. This capability guides the robot to approach these markers and align itself at predetermined critical positions. During the dental procedure setup, the stereo camera captures three-dimensional visual data of the environment, which enables the system to detect and interpret the position of the visual marker. Upon identifying the marker, the stereo camera processes the visual information and transmits it to the robot, providing instructions to navigate to the marker location. This autonomous process allows the robot to move to specific zones and execute tasks with high precision. The stereo camera’s role in environment registration extends beyond mere detection. It assists in mapping the environment by identifying the marker and registering essential points such as the drilling area, the starting point for the drilling process, the endpoint, and the drill-changing location. These predefined points are stored and used to guide the robot through the various stages of the experiment, ensuring that each task is performed in the correct sequence and at the appropriate locations. In the search for a reliable method of marker detection, ArUco markers [39] have been selected as an accurate solution (Figure 11). The principal advantage of these markers is that a single marker provides sufficient correspondence to determine the camera pose. Furthermore, the inner binary codification renders them particularly robust, enabling the application to detect errors and use correction techniques.

Upon generating the marker, the necessary nodes can be executed to determine whether the camera can detect the markers. The code included in the package is designed to employ a stereo camera configuration for the detection of ArUco markers. The principal objective of this node is to accurately identify markers in the images captured by the camera and to broadcast data regarding the identified markers for subsequent processing. Upon initialization, the ArucoSimple node configures the required ROS interfaces, including publishers, subscribers, and a transform listener. It subscribes to the camera image topic, which provides the camera calibration parameters, and the camera information topic, which supplies the images. It also publishes various items, including the pose, transform, position, and visualization markers for Rviz of the detected markers. The image callback function constitutes the core of this node. This function is triggered each time the camera receives a new image.

Additionally, the node manages rectification and camera calibration settings to ensure the accuracy of the recognized marker positions. It also offers options to fine-tune the marker detection process, such as threshold parameters and corner refinement method settings.

**Figure 12:**
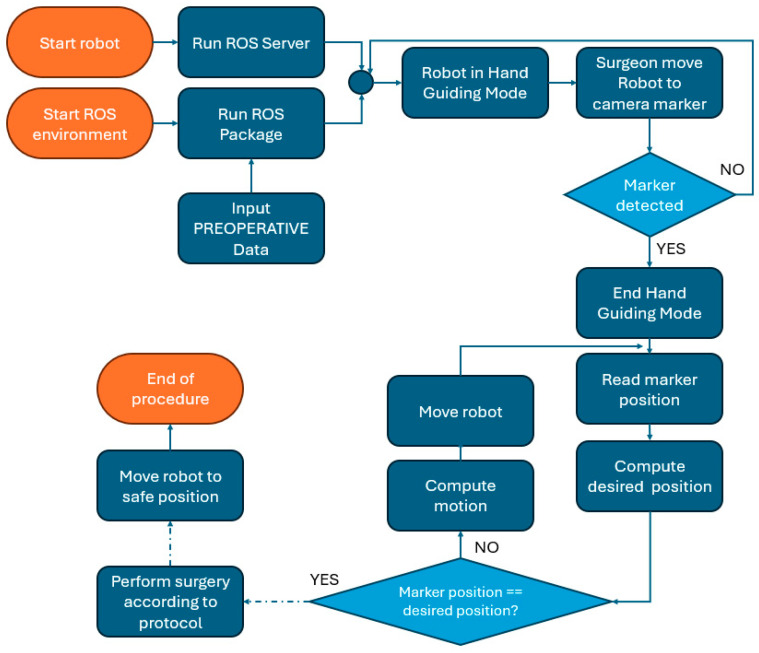
Flow chart illustrating robot actions during surgery with respect to camera marker.

For a better understanding of the control system of the robot, a simple flow chart illustrating the robot’s actions is presented in Figure 12. The robot position check using the 3D navigation camera runs on a separate thread as a background application, continuously checking differences between the robot position and the desired robot position with respect to the camera marker; if differences between the two positions are identified, the surgery procedure stops until the desired position is reached.

### 2.6. Experimental Tests

**Figure 13:**
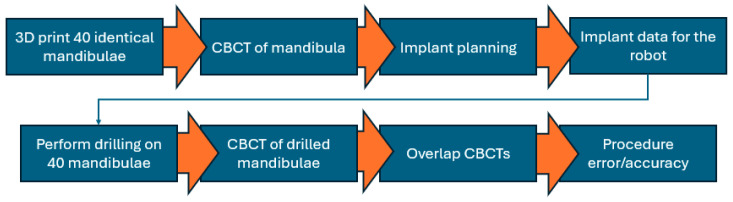
Testing plan for robotic-assisted dental implant surgery.

In order to validate the experimental setup and the medical protocol for robotic-assisted dental implant surgery, a series of experimental tests were performed. The testing plan for the robotic system is presented in Figure 13. The drilling sequence implied the first drill using a 2 mm pilot drill for cortical decortication with a depth of 10 mm using 1200 RPM speed for the drill. The second drilling was performed using a 2.8 mm drill at 800 RPM speed, followed by the final drilling using a 3.3 drill at 800 RPM speed. A 4.5 mm in diameter and 10 mm length from Megagen Anyridge [36] was inserted using torque control of the physiodispenser (maximum 30 Ncm) and 20 RPM for the wrench.

For testing the robotic system’s capability to perform accurate drilling for the implant, the number of representative samples should be determined. The accuracy study is performed only using the robotic system on 3D-printed mock-ups of mandibula and not using a control group. For sample size determination, a confidence level of 95% (*Z*-score = 1.96) was used. The robotic system is tested in terms of accuracy regarding coronal, apical, and angular deviation. Yang et al. [40] performed an accuracy assessment of robotic-assisted implant surgery in dentistry, where they determined the global standard deviation for coronal (0.6 mm), apical (0.7 mm), and angular (1.6 degrees) errors. These data are further used to determine the number of samples required for the experimental tests. To determine the number of samples, Equation (8) is used, where *Z* is the *Z*-score, σ is the standard deviation, and E is the margin of error (considered 0.5 mm).

(8) $$n=\left(\frac{Z_{\alpha /2}^{2}\cdot \sigma^{2}}{E^{2}}\right)$$

Using Equation (8), the following number of samples is determined for each type of deviation: 5.53 for coronal deviation, 7.52 for apical deviation, and 39.33 for angular deviation.

For testing, 40 identical mandibulae are 3D printed. In order to eliminate the error of the 3D printer, a CBCT is performed after the 3D printing on the mandibula mock-up. Using the later CBCT, the implant planning procedure is performed, and the data obtained are sent to the robotic system. The robot performs the drilling required for the implant on the 40 mandibulae using the same parameters. After the drilling, all 40 mandibulae are sent to the CBCT, and the initial CBCT is superposed over the later CBCT. In order to determine the error and accuracy of the procedure, the differences between the planned implant cavity and the cavity made using the robot system are analyzed.

**Figure 14:**
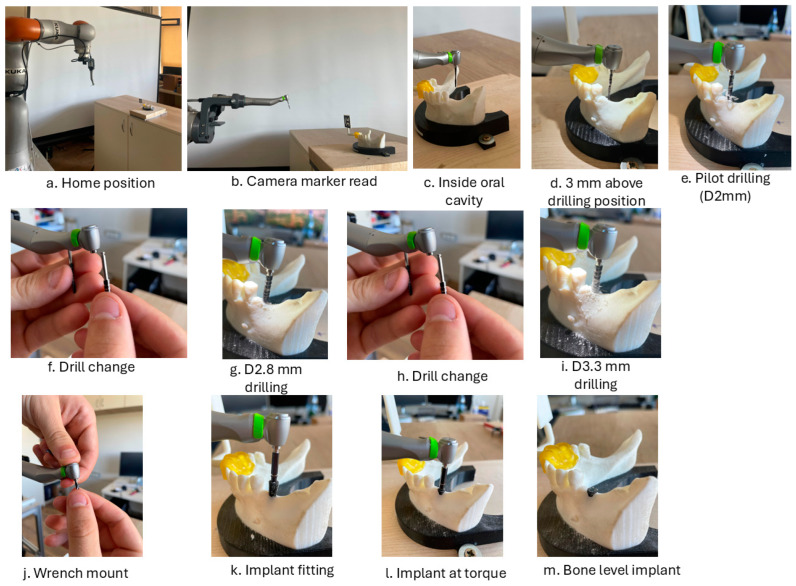
Robot positions during the surgical procedure.

The robot positions during the surgery are presented in Figure 14. The robot starts from a neutral position (Figure 14a) and is hand-guided to a position from where it can record the camera marker position (Figure 14b). The robot enters the oral cavity (Figure 14c), positions 3 mm above the insertion point (Figure 14d), and starts the pilot drilling procedure (Figure 14e). After finishing the pilot drilling procedure, the robot moves outside the oral cavity, where the dentist feeds the following drill (Figure 14f). Two sequential drillings are performed using D2.8 mm (Figure 14g) and D3.3 mm (Figure 14i) drills, and after finishing the drilling, the robot moves again outside the oral cavity where the wrench adapter is mounted in the dental handpiece (Figure 14j). Depending on the bone type, the wrench torque is set to 20–50 Ncm depending on bone density type (for type 4, 20 Ncm are used). Next, the implant is fitted (Figure 14k) and screwed (Figure 14l) until it reaches the bone level (Figure 14m).

## 3. Results

**Figure 15:**
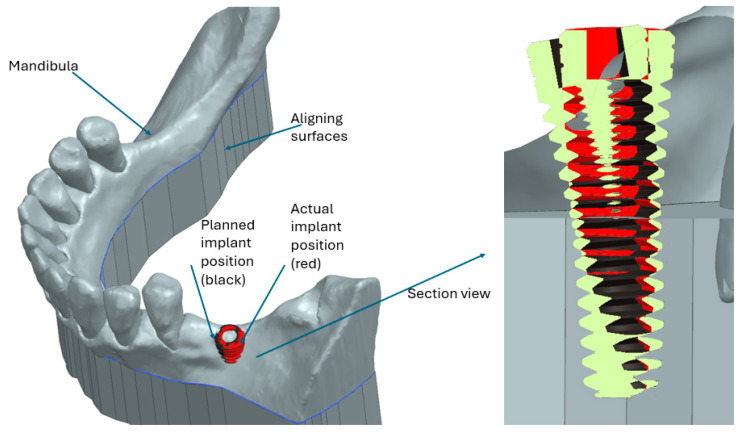
Difference between planned and actual implant (Siemens NX view [41]).

The differences between the planned implant and the actual one performed using the robotic system can be viewed in Figure 15, where an initial CBCT scan of the mandibula was superposed over a drilled mandibula; later, the implant was virtually placed after converting the CBCT scan into a 3D model, using the cavity created with the robotic system. The black color implant represents the planned implant, and the red implant represents the actual implant placed using the robot. As can be observed, the differences between the planned implant and the actual one are small (merely visible), but in order to determine the accuracy of the robotic system, these differences were measured for each of the 40 drilled mandibulae.

**Figure 16:**
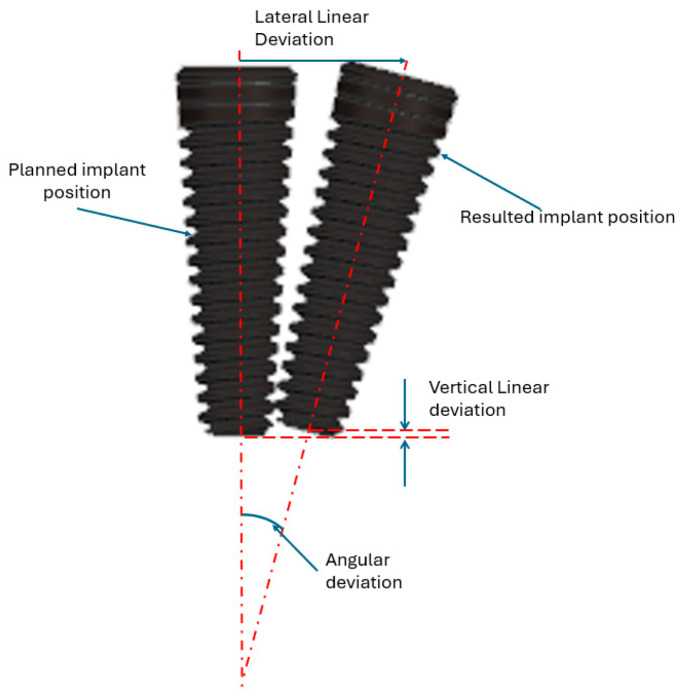
Implant deviations.

In order to measure the differences between the implants, the measurements highlighted in Figure 16 were performed for each set of mandibulae overlapping.

Table 2 presents the mean value, standard deviation, and 95% confidence level.

## 4. Discussion

Robotic-assisted dental implant placement has revolutionized the field of oral surgery by significantly improving the accuracy and reproducibility of implant positioning. Various navigation systems have emerged, each presenting unique features, advantages, and limitations. Guided surgery systems are among the most commonly employed techniques in dental implantology. Utilizing preoperative planning software, these systems create surgical guides based on three-dimensional imaging, such as cone beam computed tomography (CBCT) scans. The primary advantage of guided surgery lies in its high precision, with linear accuracy typically within 1 mm and angular accuracy ranging from 2° to 3° [42]. The clarity of the surgical roadmap reduces surgical time and enhances overall efficiency. However, despite their advantages, guided systems have a static nature; they cannot adapt to intraoperative anatomical changes. Furthermore, they necessitate a learning curve for clinicians to effectively utilize pre-surgical planning tools, which can impact the overall surgical workflow [43]. In contrast, robotic-assisted systems facilitate real-time adjustments during the procedure, allowing for dynamic adaptation to the surgical environment. Robotic systems demonstrate enhanced reproducibility, often achieving linear deviations of ≤1 mm and angular deviations of ≤1° [44]. This level of precision can improve the safety and efficacy of the procedure by accommodating real-time anatomical changes, thereby reducing the risk of complications. Nevertheless, the primary drawbacks of robotic systems include high costs and complexity. Implementation can be resource-intensive, and optimal operation requires advanced technical skills and training, which may not be readily available in all clinical settings. Another emerging category is computer-assisted systems, which encompass a range of technologies that utilize algorithms for implant placement planning and execution. These systems can vary from basic software that assists in positioning to more complex systems integrated with robotics. The advantages of computer-assisted systems lie in their enhanced planning capabilities through real-time imaging integration, enabling dynamic adjustments to implant positions. The advanced visualization features aid in informed decision-making during surgery, thus potentially improving outcomes. However, these systems’ effectiveness can be influenced by software accuracy and the clinician’s familiarity with the technology. Consequently, variability in results may occur, depending on the specific software package used. Freehand techniques, while traditional, remain prevalent in clinical practice. These methods rely heavily on the surgeon’s skill and experience for accurate implant placement. One significant advantage of freehand techniques is their accessibility; there is no need for expensive equipment or software, making them easier to implement in various clinical settings. Furthermore, these techniques allow for flexibility, enabling surgeons to adapt to unforeseen circumstances during the procedure. However, the reproducibility of freehand techniques tends to be lower than that of guided or robotic systems. Studies indicate that outcomes can exhibit high variability, with linear deviations sometimes exceeding 2 mm and angular deviations ranging from 5° to 10° [42]. The results are heavily reliant on the surgeon’s experience and expertise, which can lead to inconsistent outcomes. Dynamic Computer-Assisted Implant Surgery (dCAIS) systems offer notable advantages over traditional freehand and static-guided systems, particularly in terms of precision, adaptability, and the learning curve for novice surgeons [45]. dCAIS systems provide real-time dynamic guidance, allowing for precise implant placement by continuously adjusting to patient movements and anatomical variations. This level of adaptability is a significant benefit, especially for novice surgeons, as it reduces the potential for errors that can occur in freehand surgery or with static guides. The system’s feedback and real-time adjustments ensure greater accuracy, even in complex or challenging cases. In contrast, freehand surgery relies entirely on the surgeon’s skill and experience, which can lead to variability in outcomes, especially for less experienced surgeons. Static-guided systems offer predefined paths but lack the flexibility to adjust during the procedure, making them less effective in dynamic surgical environments. For novice surgeons, dCAIS systems shorten the learning curve by providing step-by-step guidance, improving surgical confidence, and reducing the risk of complications. Experienced surgeons also benefit from enhanced precision and reduced time in surgery. Overall, dCAIS systems offer significant improvements in accuracy, safety, and efficiency, making them a valuable tool in implant dentistry.

The robotic system proposed in this paper offers significant advantages over traditional freehand and static-guided systems. The KUKA robotic arm ensures highly precise, repeatable movements, reducing human error during the implant placement process. This precision is especially beneficial for novice surgeons, as it compensates for inexperience by guiding the surgeon in real time. In contrast, freehand surgery relies entirely on the surgeon’s skill, making it more prone to variability, particularly in less experienced hands. The integration of ROS enhances the system’s flexibility and real-time adaptability. It allows seamless communication between the robotic arm and the 3D cameras, which continuously monitor and adjust the surgical process based on real-time data. This capability ensures that the implant is placed with high accuracy, even in complex or dynamic environments. In contrast, static-guided systems only offer predetermined guidance, leaving little room for adjustment during surgery. Although these systems can provide some level of precision, they do not adapt in real time, which may limit their effectiveness, especially in cases where the patient’s anatomy or position changes during surgery. For novice surgeons, the dynamic nature of the system reduces the learning curve by providing continuous feedback, making them more confident and reducing the chances of mistakes. Even experienced surgeons benefit from the system’s high accuracy, reduced surgical time, and the ability to handle complex cases more efficiently.

The results obtained using the proposed robotic solution illustrate a higher accuracy compared to the manual procedure, being in the same range of values as other robotic systems, validating the functionality of the robotic system. The control solution was also validated, as during the 40 experiments, the robot behaved properly and without any errors. The deviation obtained after data analysis is within allowable ranges. In [46], Cunha determined the mean angular deviation for the manual procedure of 2.04°, vertical linear deviation of 0.82 mm, and lateral deviation of 0.68, while the robotic-assisted procedure proposed in this paper achieved a 21% reduction in the angular deviation, 47% reduction for the vertical linear deviation and almost 23% improvement for the lateral linear deviation. The values obtained are comparable with those of other research testing the implant deviation of robotic-assisted implants with respect to the manually placed implants.

Table 3 presents several robotic solutions that reported similar accuracy tests in dental implant surgery. By analyzing the means for angular, vertical, and lateral deviation, the robotic solution proposed within this paper scored a mean value lower than the mean value of the robotic systems analyzed, yielding the fact that the results represent an improvement compared to the manual procedure, but at the same time, the robotic the proposed solution is comparable to other robotic systems in terms of accuracy.

Accuracy in dental implant placement is crucial for the success of the procedure and the long-term health of the patient. Precise implant positioning ensures proper osseointegration, while inaccuracies (implant too deep, too shallow, wrong angle) resulting in insufficient bone–implant contact may lead to implant failure. Accuracy is also essential to avoid damage to critical anatomical structures. Implant misplacement can cause permanent nerve damage, leading to numbness, pain, or tingling sensations in the lower lip, chin, or gums, affecting the patient’s quality of life. Furthermore, in the upper jaw, implants misplaced may perforate the sinus cavity, resulting in infections. The functional outcomes of the implant are also greatly influenced by its accuracy. Proper implant placement ensures that the prosthesis (such as crowns or bridges) fits perfectly with adjacent teeth and aligns properly with the opposing dentition. A misaligned implant can lead to issues with occlusion—the way the upper and lower teeth meet—which can cause discomfort, uneven bite pressure, or even damage to the implant or surrounding teeth. Moreover, an improperly placed implant can lead to aesthetic issues, especially in the anterior (front) region of the mouth, where implant position affects both the appearance and symmetry of the smile.

Finally, the precision of the implant affects the long-term durability of both the implant and the prosthetic restoration. The more accurate the placement, the less stress is placed on the implant and surrounding tissues, reducing the chances of complications such as implant failure or bone loss over time. Accurate implant placement reduces the need for revision surgeries or additional interventions, which not only improves patient outcomes but also reduces healthcare costs in the long term.

Considering the success rate of the implant being almost 98%, an excellent success rate, the 2% failure rate can still have significant clinical consequences. Even a small reduction in the likelihood of failure can have a substantial impact on a large number of procedures. The improved accuracy offered by robotic systems minimizes errors such as misalignment, incorrect depth, or angulation, which are often the primary causes of implant failure or complications. For instance, a slight deviation in placement can lead to poor osseointegration, nerve damage, or damage to surrounding anatomical structures, increasing the risk of complications, which may not be fully captured by an overall success rate. Reducing the failure rate, even by a small margin, helps to ensure that implants remain stable and function properly over the long term, reducing the need for corrective procedures or replacements. There is no clear statistic revealing the number of dental implants performed annually. The reported data vary between 1.8 and 3 million implants placed annually. By improving the success rate of the procedure by only 1 percent, the number of successful implants will rise by over 18.000, considering that the cost of an implant varies between EUR 1500 and 6000, resulting in a cost reduction of EUR 27 to 108 million.

While acknowledging the lower number of tests compared to other studies, the experimental data allow an initial validation of the proposed robotic system, emphasizing the need for future improvements.

There are still some aspects to be clarified regarding the navigation system and the force/torque monitoring system.

The navigation system uses a commercial stereo camera that has high precision and close-range depth-sensing capabilities. It has a depth accuracy of approximately 0.1% of the distance from the camera to the object. This means that for an object 1 m away, the depth measurement accuracy is around 1 mm. The camera has an optimal depth range of 7 to 50 cm, a resolution of 1280 × 720 pixels for depth, 1920 × 1080 pixels for RGB, and is able to record up to 90 frames per second. During the experimental testing, the positioning results were partially influenced by the noise in camera reading and the robot vibration during the motion, which are topics for further investigation. Future work includes experimental and analytical determination of robot vibrations and the use of several denoising filters of the camera input to obtain more accurate values. New experiments will be performed on anatomic models that will allow the positioning of the mandibulae at different angles and will include a complete anatomic model of the oral cavity, which also integrates the upper jaw.

During the experiments, the force/torque monitoring system integrated within the Kuka iiwa 7 robot was used. Even though the system is capable of collaborative tasks, the force monitoring system during the drilling was not sensitive enough for force values of less than 10 Newtons. In future work, an external force sensor will be employed to address this issue.

Another limitation refers to the patient motion tracking, which is currently performed using real-time feedback from the visual marker, but for sudden motions, a delay of 0.1 to 0.8 s was recorded, which is unsuitable in a real-case scenario. To address this issue, future work in the development of robotic surgical systems should focus on the integration of predictive algorithms that can anticipate the patient’s future head movements. By leveraging artificial intelligence (AI) and machine learning (ML), these algorithms can analyze historical motion data and predict short-term movements before they occur. Such predictive capabilities would enable the system to adjust in real time, compensating for sudden shifts in the patient’s position and reducing the impact of motion delays.

Incorporating AI-based predictive tracking could significantly improve the accuracy and efficiency of robotic systems by ensuring that the system’s feedback loop is faster and more anticipatory. With these advancements, the robotic system would not only correct for current motion but also prepare for imminent changes, resulting in smoother and more accurate surgical outcomes. This innovation would make robotic surgery more reliable and clinically effective, particularly in complex procedures where high precision is essential for patient safety and recovery.

During the experimental tests, it was observed that the marker holder guide, developed as a single, multi-material part, had no negative influence regarding the positioning accuracy, its main purpose being to support the marker holder for the navigation camera, which had a fixed position with respect to the mandible. Future work implies a new approach, aiming to remove entirely the marker holder guide and the implementation of virtual surgical guides by combining, in augmented reality, real-time visual data with CBCT scans and the use of anatomic structures as registration markers between the robot and the patient.

The null hypothesis was rejected because significant deviations were observed between the positions of the planned and placed implants when utilizing the proposed robotic system. This study found that there were measurable differences in both linear and angular placements of the implants. Specifically, the discrepancies indicated that the actual implant positions did not align with the intended positions derived from the preoperative planning. Such variations suggest that the robotic system, while effective, does not guarantee perfect accuracy in implant placement. The findings highlight the importance of evaluating the precision of guided systems in clinical practice. These results contribute to the ongoing discussion regarding the reliability of navigation technologies in dental implantology. As a result, further investigations are warranted to enhance the precision of these systems. Overall, the rejection of the null hypotheses underscores the need for continuous improvement in surgical planning technologies. This research emphasizes the complexity of achieving optimal outcomes in dental implant placement.

The initial experimental results encourage the further development of the robotic system. The use of ROS enables simple and fast integration of new components, which enables the development of new 3D navigation solutions, integration of additional sensing systems, and new software modules to enhance the robot–patient interaction in terms of procedural efficiency and safety.

The field of robotic-assisted dental implant placement faces several limitations that warrant consideration. One major challenge is the high cost of advanced technologies, which can restrict access to many dental practices and limit widespread adoption. Using medically approved robots is more cost-effective in the short term because they are already developed, tested, and integrated into clinical practice. The initial investment involves acquisition costs, but these robots come with established support, proven outcomes, and optimized performance, reducing the risk of technical issues. In contrast, designing new robots requires significant R&D, clinical trials, and regulatory approval, leading to higher upfront costs and longer implementation periods. While new robots may offer advanced features, they carry a higher financial risk and a longer timeline to achieve cost savings.

Moreover, variability in research outcomes presents a challenge in establishing consistent standards for these technologies. Different study designs, sample sizes, and measurement methods can yield inconclusive findings, making it difficult to draw definitive conclusions about the effectiveness of robotic systems. Furthermore, the success of guided systems and robotic technologies heavily relies on the accuracy of preoperative planning, as inaccuracies in imaging or planning can lead to discrepancies in implant placement.

Looking toward the future, technological advancements are expected to play a crucial role in addressing these limitations. Continued innovations may lead to the development of more affordable and user-friendly robotic systems, increasing accessibility for practitioners. Integrating artificial intelligence and machine learning could further enhance the precision of implant placement by enabling real-time adjustments based on intraoperative feedback. Additionally, ongoing research into improving preoperative planning techniques and imaging accuracy is essential for maximizing the benefits of robotic-assisted systems in clinical practice.

## 5. Conclusions

This paper introduces an innovative robotic system for implant surgery that integrates a collaborative robot with an end-effector-mounted 3D navigation camera designed to autonomously perform implant site drilling. The system builds upon traditional manual procedures, introducing a new medical protocol that includes a preplanning phase to optimize the surgery. The system was experimentally tested on 40 3D-printed mandibulae. Prior to drilling, the mandibulae were scanned using CBCT, and the 3D models were compared against those scanned post-implant placement. The spatial positioning accuracy of the implant was assessed, with the highest error recorded in angular deviation (1.62° ± 0.21°), followed by lateral linear deviation (0.53 mm ± 0.11 mm) and vertical linear deviation (0.44 mm ± 0.08 mm).

The practical benefits of this robotic system are particularly evident for different user groups. For novice surgeons, the system provides increased guidance and precision, reducing the risk of errors associated with manual procedures and thus shortening the learning curve. For experienced surgeons, the system offers enhanced accuracy and efficiency, enabling them to handle more complex cases with greater confidence and precision. The integration of robotic assistance could ultimately improve patient outcomes by ensuring more precise implant placement, potentially reducing complications and recovery time.

Looking forward, future work will focus on the development of a dynamic 3D navigation system, replacing static guides with virtual ones. This will involve the use of augmented reality to fuse real-time images with CBCT data, along with the use of anatomic markers for patient–robot registration. These advancements aim to improve real-time adaptability and further streamline the surgical process. The next steps for clinical application involve refining these technologies to ensure they are ready for broader adoption in clinical settings, with continued research focusing on enhancing the system’s accuracy and usability in real-world surgeries.

## Figures and Tables

**Table 1:** Feed rate parameters based on bone density.

Classification	*N*	$\rho_{a}$
D1	800–1000	1–2
D2	1000–1200	0.5–1
D3	1200–1500	0.2–0.5
D4	1500+	0.1–0.2

**Table 2:** Measurements after drilling the mandibulae.

Measurement	Mean Value	Standard Deviation	95% Confidence Lower Interval	95% Confidence Upper Interval
Angular deviation [°]	1.62	0.67	1.30	1.73
Lateral linear deviation [mm]	0.53	0.36	0.42	0.64
Vertical linear deviation [mm]	0.44	0.26	0.35	0.52

Analyzing the measurements of the experiment, the highest error was obtained in the angular deviation: 1.62° ± 0.21°, followed by lateral linear deviation: 0.53 mm ± 0.11 mm and vertical linear deviation: 0.44 mm ± 0.08 mm.

**Table 3:** Results obtained by other robotic systems for oral surgery.

Study	Angular Deviation	Lateral Linear Deviation	Vertical Linear Deviation	Number of Implants
Thangwarawut [47]	1.49 ± 0.53°	0.34 ± 0.16 mm	0.19 ± 0.13 mm	72
Xu [48]	0.61 ± 0.25°	0.29 ± 0.15 mm	0.29 ± 0.15 mm	216
Tian-shu [49]	3.74 ± 0.67°	1.04 ± 0.37 mm	1.56 ±0.52 mm	15
Bolding [50]	2.56 ± 1.48°	1.04 ± 0.7 mm	0.95 ±0.73 mm	38
Yang [18]	1.11 ± 0.46°	0.74 ± 0.29 mm	0.73 ±0.28 mm	10
Chen [51]	1.08 ± 0.66°	0.58 ± 0.31 mm	0.69 ±0.28 mm	10
Chen [51]	2.32 ± 0.71°	0.73 ± 0.20 mm	0.86 ±0.33 mm	10
Shi [52]	3.46 ± 3.11°	1.17 ± 0.36 mm	1.41 ±0.62 mm	20
Mean	2.04 ± 1.15°	0.74 ± 0.32 mm	0.83 ±0.48 mm	

## Data Availability

The raw data supporting the conclusions of this article will be made available by the authors on request.
